# Structure and Polymorphism of the Major Histocompatibility Complex Class II Region in the Japanese Crested Ibis, *Nipponia nippon*

**DOI:** 10.1371/journal.pone.0108506

**Published:** 2014-09-23

**Authors:** Yukio Taniguchi, Keisuke Matsumoto, Hirokazu Matsuda, Takahisa Yamada, Toshie Sugiyama, Kosuke Homma, Yoshinori Kaneko, Satoshi Yamagishi, Hiroaki Iwaisaki

**Affiliations:** 1 Laboratory of Animal Breeding and Genetics, Graduate School of Agriculture, Kyoto University, Kyoto, Japan; 2 Department of Agrobiology, Faculty of Agriculture, Niigata University, Niigata, Japan; 3 Field Center for Sustainable Agriculture and Forestry, Niigata University, Niigata, Japan; 4 Sado Japanese Crested Ibis Conservation Center, Sado, Japan; 5 Yamashina Institute for Ornithology, Abiko, Japan; CSIRO, Australia

## Abstract

The major histocompatibility complex (MHC) is a highly polymorphic genomic region that plays a central role in the immune system. Despite its functional consistency, the genomic structure of the MHC differs substantially among organisms. In birds, the MHC-B structures of Galliformes, including chickens, have been well characterized, but information about other avian MHCs remains sparse. The Japanese Crested Ibis (*Nipponia nippon*, Pelecaniformes) is an internationally conserved, critically threatened species. The current Japanese population of *N. nippon* originates from only five founders; thus, understanding the genetic diversity among these founders is critical for effective population management. Because of its high polymorphism and importance for disease resistance and other functions, the MHC has been an important focus in the conservation of endangered species. Here, we report the structure and polymorphism of the Japanese Crested Ibis MHC class II region. Screening of genomic libraries allowed the construction of three contigs representing different haplotypes of MHC class II regions. Characterization of genomic clones revealed that the MHC class II genomic structure of *N. nippon* was largely different from that of chicken. A pair of MHC-IIA and -IIB genes was arranged head-to-head between the *COL11A2* and *BRD2* genes. Gene order in *N. nippon* was more similar to that in humans than to that in chicken. The three haplotypes contained one to three copies of MHC-IIA/IIB gene pairs. Genotyping of the MHC class II region detected only three haplotypes among the five founders, suggesting that the genetic diversity of the current Japanese Crested Ibis population is extremely low. The structure of the MHC class II region presented here provides valuable insight for future studies on the evolution of the avian MHC and for conservation of the Japanese Crested Ibis.

## Introduction

The major histocompatibility complex (MHC) is a highly polymorphic genomic region that plays a central role in the immune system of all jawed vertebrates. The MHC class I and class II genes encode glycoproteins that transport foreign peptides to the surfaces of cells for recognition by T-cell receptors on lymphocytes, which in turn triggers the adaptive immune response [1]. Therefore, this genomic region is crucial for resistance and susceptibility to pathogenic disease. Polymorphisms at MHC class I and class II genes facilitate binding of a diversity of pathogens, and these evolutionary selection pressures are thought to contribute to the high genetic variation in MHC loci [2]. The MHC class II molecule is a heterodimer consisting of an α and a β chain, which are encoded by MHC-IIA and -IIB genes, respectively.

Polymorphism in the MHC is not restricted to allelic variation. The molecular evolution of the MHC involves frequent gene duplication and gene loss that result in vast rearrangements and pronounced variation in gene number and genomic organization among organisms [3], [4]. In birds, two MHC-IIB lineages (*DAB1* and *DAB2*) have been characterized [5]. Phylogenetic reconstructions and simulations using 63 MHC-IIB exon 3 sequences from six avian orders have suggested that a unique duplication event preceding the major avian radiations gave rise to ancestral MHC-IIB lineages that were each likely lost once later during avian evolution [6]. However, to obtain deeper insights into the long-term evolutionary history of the avian MHC, more data from other exons, other genes and ultimately genomic structures are required.

The domestic chicken (*Gallus gallus*, order Galliformes) has been most intensively studied and its MHC (also known as MHC-B or B-complex) has a remarkable structure referred to as a “minimal essential MHC” [7]. In contrast to the human MHC (human leukocyte antigen, HLA), which spans approximately 7.6 Mb and contains 421 gene loci on a contiguous region [8], the chicken MHC-B consists of only 19 genes spanning 92 kb [7]. In addition to MHC-B, chicken MHC class I and class II genes are present in a separate and unlinked cluster called the MHC-Y region [9], [10].

The overall MHC-B structures of five other galliform species, Japanese Quail (*Coturnix coturnix japonica*), domestic turkey (*Meleagris gallopavo*), Golden Pheasant (*Chrysolophus pictus*), Black Grouse (*Tetrao tetrix*), and Greater Prairie-Chicken (*Tympanuchus cupido*) are largely similar to that of chicken, whereas gene number, order, and orientation in these structures vary among the species [11]–[15].

However, studies of non-galliform species, such as duck (*Anas platyrhynchos*, order Anseriformes) and Zebra Finch (*Taeniopygia guttata*, order Passeriformes) have suggested that the chicken minimal essential MHC is not typical among birds [16]–[18]. For example, the chicken MHC-B contains two MHC-IIB genes (*BLB1* and *BLB2*) but no MHC-IIA genes (*BLA*), whereas the duck MHC possesses one MHC-IIA and five MHC-IIB genes [16]. Because taxonomic and genomic sampling of avian MHC regions is limited, it is unclear whether the minimal essential MHC represents the ancestral condition or whether it is a highly derived condition unique to the Galliformes.

The Japanese Crested Ibis (*Nipponia nippon*, order Pelecaniformes) is an internationally conserved bird, listed as “Endangered” in the 2012 International Union for Conservation of Nature Red List of Threatened Species (http://www.iucnredlist.org). The range of *N*. *nippon* formerly included much of Japan and northeastern Asia, but habitat loss and overhunting for its feathers have caused a drastic decline in its numbers and resulted in its extinction in Japan. Captive-breeding programs have been conducted using five birds as founders (two individuals introduced in 1999, one introduced in 2000, and two introduced in 2007) provided by the Chinese government. The current size of the captive-breeding population in Japan is approximately 210 birds, most of which are on Sado Island. The Ministry of the Environment of Japan launched a project to release *N*. *nippon* on Sado Island in 2008; in April 2012, three chicks hatched there, the first of this species born in the wild in 36 years [19].

Molecular ecology studies have shown that, in addition to adaptive immune responses, the MHC genotype influences patterns of mate choice, local adaptation, and expression of sexually selected ornaments [20]–[23]. For these reasons, the diversity of MHCs is of major interest to the conservation of endangered species.

The Japanese Crested Ibis belongs to an avian lineage that is highly divergent from that of chicken, duck, or Zebra Finch. Characterizing the MHC class II region in *N*. *nippon* may provide valuable information about the primordial avian MHC. In addition, insight into the genetic diversity of this genome region could be vital to conservation of the *N*. *nippon* population. In this study, we investigated the genomic organization of the *N*. *nippon* MHC class II region and polymorphisms among the five founders of the Japanese population.

## Materials and Methods

### Samples

Blood samples from *N*. *nippon* (five founders, A–E, and 20 progeny) and the liver from a dead female were provided by the Sado Japanese Crested Ibis Conservation Center (Niigata, Japan). Sample-collection protocols were based on a conservation project of the Ministry of the Environment of Japan and approved by the Animal Research Committee of Niigata University. Genomic DNA samples were prepared from whole blood and liver using the Wizard Genomic DNA Purification kit (Promega) according to the manufacturer’s instructions.

### Primers

**Figure 1:**
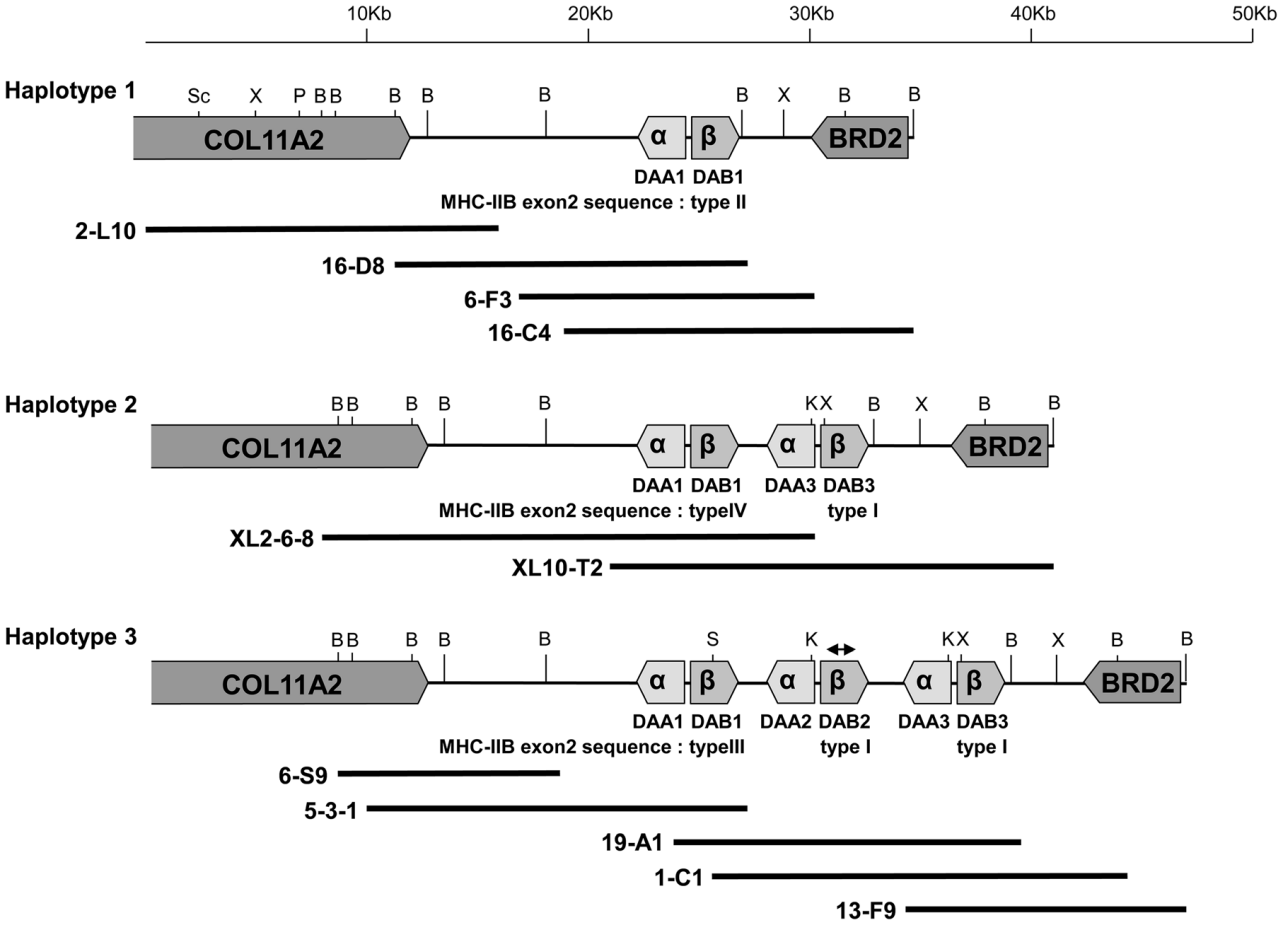
Genomic organization of the Japanese Crested Ibis MHC class II region. Three contigs representing different haplotypes were constructed. *Collagen-type XI α-2* (*COL11A2*), MHC-IIA (*α*), MHC-IIB (*β*) and *bromodomain-containing 2* (*BRD2*) genes and their orientations are indicated. Locus names are indicated below the MHC-IIA and -IIB genes. The types of MHC-IIB exon 2 sequences (Table 1) are shown below the locus names. B, K, P, S, Sc, and X represent restriction sites used for subcloning of *Bam*HI, *Kpn*I, *Pst*I, *Sal*I, *Sac*I, and *Xho*I, respectively. Solid bars below the map represent locations of isolated lambda phage clones. The bidirectional arrow above *DAB2* in haplotype 3 indicates the first amplified MHC-IIB fragment.

Primers and annealing temperatures used for polymerase chain reaction (PCR) analysis are shown in Table S1. To determine the partial sequence of the Japanese Crested Ibis MHC class IIB gene, degenerate primers BRMHC05 and AIEx3R (Table S1) [24], [25] were used for amplification and an amplified 1,057 bp fragment containing exon 2 was cloned and then sequenced. The resulting sequence represented a part of the *DAB2* locus (Figure 1). Primers 2F_pen1 and intron2-01R (Table S1) were designed on the basis of a previous report [26] and the sequence of this PCR product. Other primers were designed on the basis of sequences determined in this study.

### Polymorphisms and PCR-restriction fragment length polymorphism (RFLP) of MHC-IIB Exon 2

A 279 bp fragment of MHC-IIB exon 2 was amplified using primers 2F_pen1 and intron2-01R (Table S1) from two founders (D and E) and 20 progeny (At the time, three founder samples, A–C were not available to us). In order to analyze polymorphism, PCR products were cloned using the TOPO TA Cloning Kit (Invitrogen) and at least 8 positive clones were sequenced per individual. Based on the sequence information of these clones, we identified possible restriction sites for further PCR-RFLP analysis and chose the restriction enzymes *Pst*I, *Rsa*I, or *Sal*I. Digested fragments were separated on a 3% agarose gel and revealed individual banding patterns for each of the different MHC-IIB exon 2 sequences. MHC class II genotypes of five founders were examined by PCR-RFLP.

### Construction of a genomic library and Screening

To determine the genomic structure of the *N. nippon* MHC class II region, two genomic libraries were constructed from a dead female and founder E. Genomic DNA was partially digested with *Sau*3AI and separated on a 0.5% agarose gel. Digested fragments (15–23 kb) were gel purified using the Wizard SV Gel and PCR Clean-Up system (Promega), according to the manufacturer’s instructions, and ligated into the Lambda DASH II vector (Stratagene). The ligated DNA mixture was then packaged using Gigapack III Gold or XL packaging extract (Stratagene). Screening was performed using a PCR-based method (Figure S1). Five lambda phage clones (16-D8, 6-F3, 5-3-1, 19-A1 and 1-C1) were isolated by the PCR-screening with primers 2F_pen1 and intron2-01R (Figure 1 and Table S1). On the basis of the sequences of both ends in two lambda phage clones (16-D8 and 1-C1), primers 16-D8Rev_S1, 16-D8Rev_A1, 16-D8Uni_S1, 16-D8Uni_A1, 1-C1BRD2_S1 and 1-C1BRD2_A1 (Table S1) were designed and used for genome walking. Two lambda phage clones (2-L10 and 6-S9) were isolated by the PCR-screening with 16-D8Uni_S1/A1 primer pair, as were a clone 6-F3 with 16-D8Rev_S1/A1 primer pair and two clones (16-C4 and 13-F9) with 1-C1BRD2_S1/A1 primer pair (Figure 1). These positive clones were isolated from the genomic library constructed from a dead female. In addition, two positive clones (XL2-6-8 and XL10-T2) were isolated from the founder E genomic library by the PCR-screening with primers 2F_pen1 and intron2-01R (Figure 1 and Table S1).

### Analysis of isolated lambda phage clones

Phage DNAs of positive clones were purified, digested with *Bam*HI and/or *Eco*RI, and subcloned into pBluescript II vector (Stratagene). In some lambda phage clones, additional restriction enzymes (*Kpn*I, *Sac*I, *Sal*I and/or *Xho*I) were also used (Figure 1). Sequences of both ends of some subclones were determined and analyzed through homology searches using BLAST (http://blast.ncbi.nlm.nih.gov/blast). Moreover, subclones containing MHC-IIB exon 2 were identified by colony-PCR with primers 2F_pen1 and intron2-01R (Table S1) and analyzed by PCR-RFLP for exon 2 sequence. By combining with these results, restriction maps of each lambda phage clones were constructed.

For complete sequencing of MHC class II genes, insert DNA of subclones containing MHC class II genes was completely or partially digested with *Sac*I or *Sau3*AI and the resulting fragments were re-cloned into pBluescript II vector (Stratagene). Positive clones were sequenced using M13 forward and reverse primers. The remaining gaps were filled by sequencing with specific primers. Every nucleotide position was sequenced at least twice using both strands of a single subclone or the same strand of several subclones. Sequencing was carried out by Greiner Japan Co., Ltd. (Tokyo, Japan). Sequences were manually assembled using GENETYX version 11 (Software Development). The sequences of MHC class II gene regions from haplotypes 1, 2, and 3 (approximately 8, 13, and 18 kb, respectively) were deposited in the DNA Data Bank of Japan (DDBJ) under accession numbers AB872442, AB872443, and AB872444, respectively. The sequence of the *BRD2* gene in haplotype 1 was deposited in the DDBJ under accession number AB890383. The partial sequence of *COL11A2*-like gene is shown in Figure S2.

### Gene Identification and Sequence Analysis

The assembled sequences were analyzed for coding regions using the GENSCAN program (http://genes.mit.edu/GENSCAN.html) with vertebrate parameters and through homology searches using BLAST (http://blast.ncbi.nlm.nih.gov/blast). Sequence alignments and dot-matrix analyses were performed using GENETYX with the default parameters.

### Construction of Phylogenetic Tree

Phylogenetic relationships of MHC-IIB genes within Pelecaniformes as well as among avian orders were analyzed using exon 2 or partial exon 3 (first 128 bp) sequences, since only 662 bp sequences containing full exon 2, intron 2 and partial exon3 are available in Pelecaniformes except for the Japanese crested ibis [27]. Eleven Pelecaniformes and two species from each of seven avian orders used for the analyses were shown in Table S3. The best-fitting nucleotide substitution model for each codon position was evaluated using Find Best DNA/Protein Models (ML) in MEGA version 5.2 [28] according to the Akaike information criterion. A maximum-likelihood tree with exon 2 sequences was constructed by using a Kimura 2-parameter model with gamma distribution in MEGA. A maximum-likelihood tree with partial exon 3 sequences was constructed by using a Tamura 3-parameter model with gamma distribution in MEGA.

### Southern blotting

In order to confirm haplotype identity for all founder individuals and to examine a presence of additional MHC loci, we performed Southern blotting. Genomic DNA (10 µg) from the five founders was digested with *Bam*HI and *Eco*RI and separated on a 0.7% agarose gel. These restriction enzymes were chosen on the basis of restriction maps and sequences of three haplotypes and preliminary experiments. The gel was blotted onto Hybond-N+ nylon membrane (GE Healthcare) and immobilized by UV cross-linking. Three fragments (238 bp of MHC-IIA exon 3, 279 bp of MHC-IIB exon 2, and 307 bp of MHC-IIB exon 3) were used as probes for the detection of MHC-IIA/IIB fragments. The probes were labeled using a PCR DIG Probe Synthesis Kit (Roche) with primers IIAex3-F and IIAex3-R for MHC-IIA exon 3, 2F_pen1 and intron2-01R for MHC-IIB exon 2, and IIBex3-F and IIBex3-R for MHC-IIB exon 3 (Table S1). Hybridization and detection were performed according to the manufacturer’s instructions.

## Results

### Polymorphism of MHC-IIB Exon 2

To determine the polymorphism of the Japanese Crested Ibis MHC class IIB gene, 279 bp of exon 2 sequences were amplified from two founders (D and E) and 20 progeny. Sequencing of the PCR products revealed four types of exon 2 sequences (temporally named types I–IV for the 279 bp exon 2 sequences), suggesting that the *N. nippon* population contained at least four alleles of the MHC class IIB gene (see below). Each individual possessed one to three exon 2 sequences (Table S2). We developed a PCR-RFLP method to distinguish the type (I–IV) of MHC-IIB exon 2. The four sequences of exon 2 could be assigned to types by the combinations of profiles digested with restriction enzymes of *Pst*I, *Rsa*I, or *Sal*I (Table 1).

**Table 1:** Digestion profiles of 279 bp of the MHC-IIB exon 2 sequence.

Exon 2 sequence	Restriction enzyme		
	*Pst*I	*Rsa*I	*Sal*I
Type I	279 bp	124 bp, 54 bp, 101 bp	279 bp
Type II	279 bp	279 bp	279 bp
Type III	279 bp	124 bp, 54 bp, 101 bp	201 bp, 78 bp
Type IV	168 bp, 111 bp	279 bp	279 bp

### Genomic Structure of MHC Class II Region

To determine the genomic structure of the *N. nippon* MHC class II region, we screened a genomic library constructed from the liver of a dead female and assembled two contigs (Figure 1). Restriction mapping and partial sequencing suggested that the contigs represented two different homologous chromosomes. One contig (approximately 35 kb) contained a partial copy of *COL11A2*, one copy of an MHC IIA/IIB pair (DAA1*01/DAB1*01), and *BRD2*; this contig was designated haplotype 1 (HP1). (Note: the *DAA1/DAB1*-nomenclature describes an allele of the full gene.) PCR-RFLP analysis of the isolated clone revealed that the exon 2 sequence of the DAB1*01 allele in HP1 was type II. Another contig (approximately 40 kb) contained a partial copy of *COL11A2*, three copies of MHC IIA/IIB pairs (DAA1*03/DAB1*03, DAA2*03/DAB2*03, and DAA3*03/DAB3*03), and *BRD2*; this contig was designated haplotype 3 (HP3). The exon 2 sequences of the DAB1*03, DAB2*03, and DAB3*03 alleles in HP3 were types III, I, and I, respectively.

To isolate a haplotype containing type IV of MHC-IIB exon 2, an additional genomic library was constructed from founder E, in which type IV was revealed by the sequencing of exon 2 PCR products (Table S2). Two positive clones were isolated and a contig (approximately 35 kb) was assembled and designated as haplotype 2 (HP2). HP2 contained a partial copy of *COL11A2*, two copies of MHC IIA/IIB pairs (DAA1*02/DAB1*02 and DAA3*02/DAB3*02), and *BRD2* (Figure 1). The exon 2 sequences of DAB1*02 and DAB3*02 alleles in HP2 were types IV and I, respectively. The names of the three MHC IIA/IIB pairs in HP3 were assigned according to the proposal for naming vertebrate MHC genes suggested by Klein et al. [29]. Names of MHC-IIA and -IIB genes in HP1 and HP2 were assigned based on homology with genes in HP3. MHC-IIA and -IIB genes were arranged head-to-head in all pairs.

**Figure 2:**
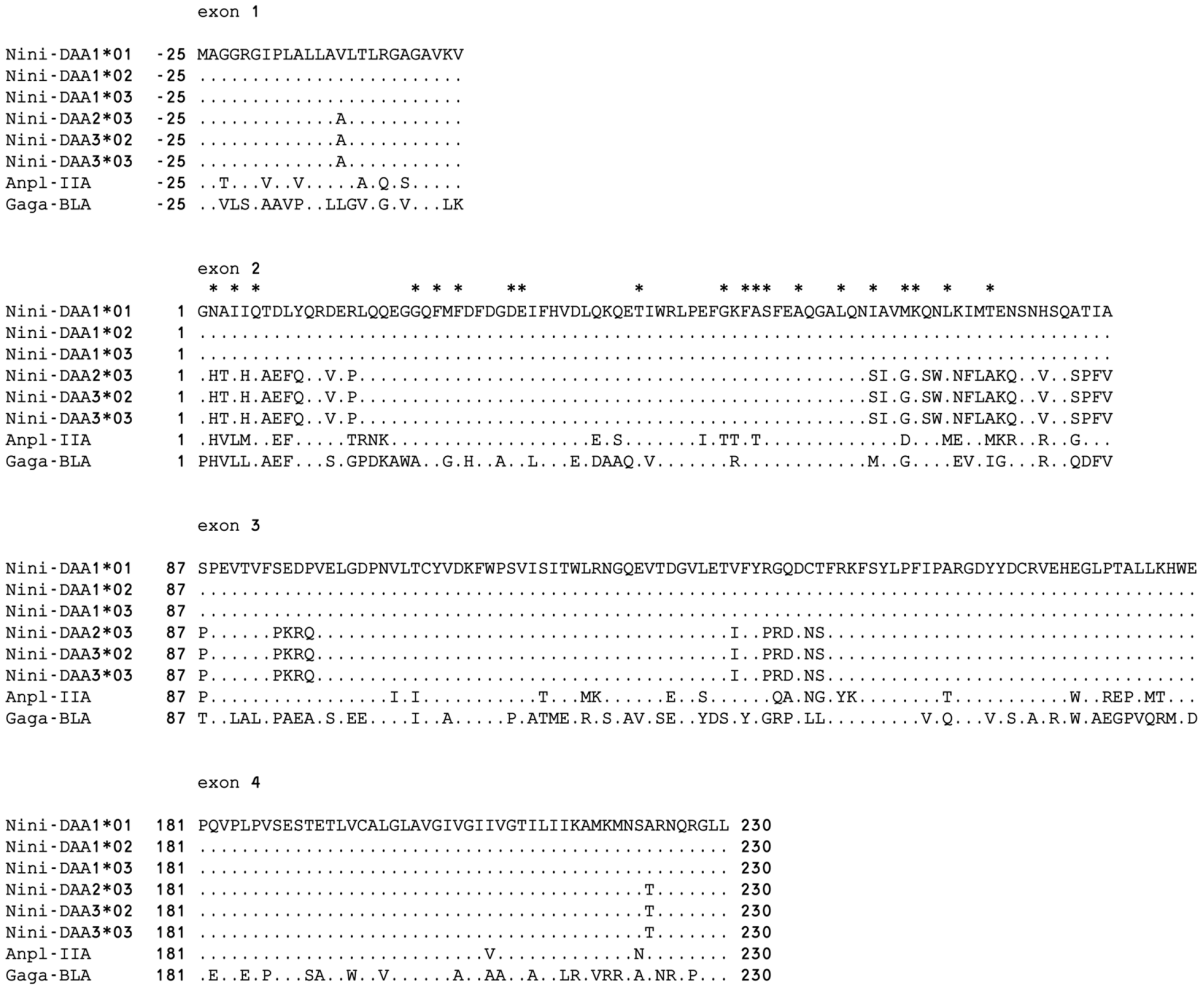
Alignment of predicted amino acid sequences of six MHC class IIA alleles from three haplotypes. The duck (Anpl-IIa) and chicken (Gaga-BLA) amino acid sequences are provided for reference. The first amino acid of the α1 domain was designated as position 1. Identity with the Nini-DAA1*01 sequence is indicated with a dot. Gaps are indicated by dashes. Asterisks above the sequence of the α1 domain indicate peptide-binding residues [39].

**Figure 3:**
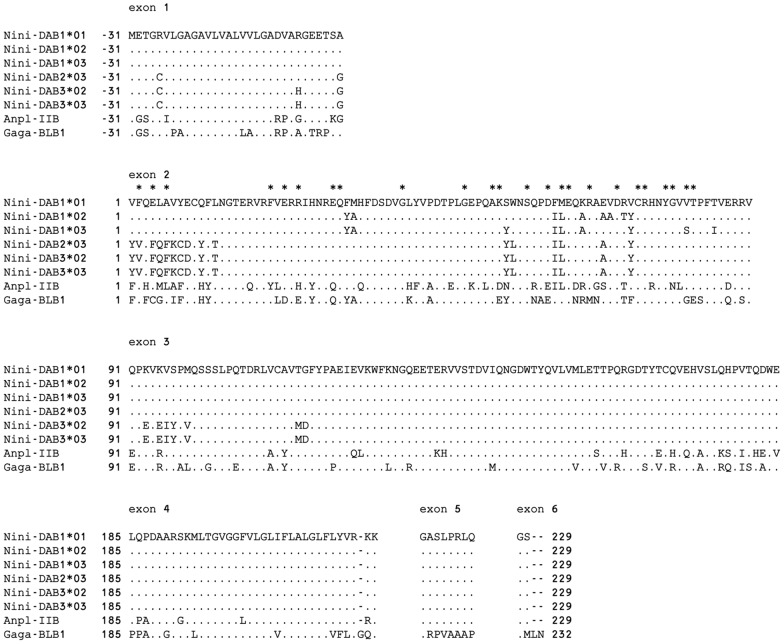
Alignment of predicted amino acid sequences of six MHC class IIB alleles from three haplotypes. Duck (Anpl-IIb) and chicken (Gaga-BLB1) amino acid sequences are provided for reference. The first amino acid of the β1 domain was designated as position 1. Identity with the Nini-DAB1*01 sequence is indicated with a dot. Gaps are indicated by dashes. Asterisks above the sequence of the β1 domain indicate peptide-binding residues [40].

To characterize the MHC-IIA and -IIB genes in detail, the complete sequences of *Bam*HI fragments (approximately 8, 13, and 18 kb from HP1, HP2, and HP3, respectively) were determined. The coding sequences in MHC-IIA and -IIB genes were predicted using GENSCAN and deduced amino acid sequences were then aligned (Figures 2 and 3). Frameshift mutations or premature stop codons were not detected in predicted MHC-IIA and -IIB genes, suggesting that every MHC class II gene could encode functional proteins. In the MHC-IIA genes, the DAA1*01, DAA1*02, and DAA1*03 alleles encoded identical proteins. Another three alleles (DAA3*02, DAA2*03, and DAA3*03) also encoded identical proteins. No single-nucleotide polymorphism (SNP) sites were observed in exons within either of the two gene groups whereas two SNP sites were detected in intron 1 of the *DAA1* locus and one SNP site was found in the promoter region of the *DAA3-DAB3* locus. In the MHC-IIB genes, the DAB3*02 and DAB3*03 alleles encoded identical proteins. Although the DAB2*03 allele was very similar to *DAB3* genes, one amino acid substitution was detected in exon 1 and seven were detected in exon 3. The *DAB1* loci (DAB1*01–*03) were highly conserved; 12 polymorphic amino acid residues were detected only within exon 2.

**Figure 4:**
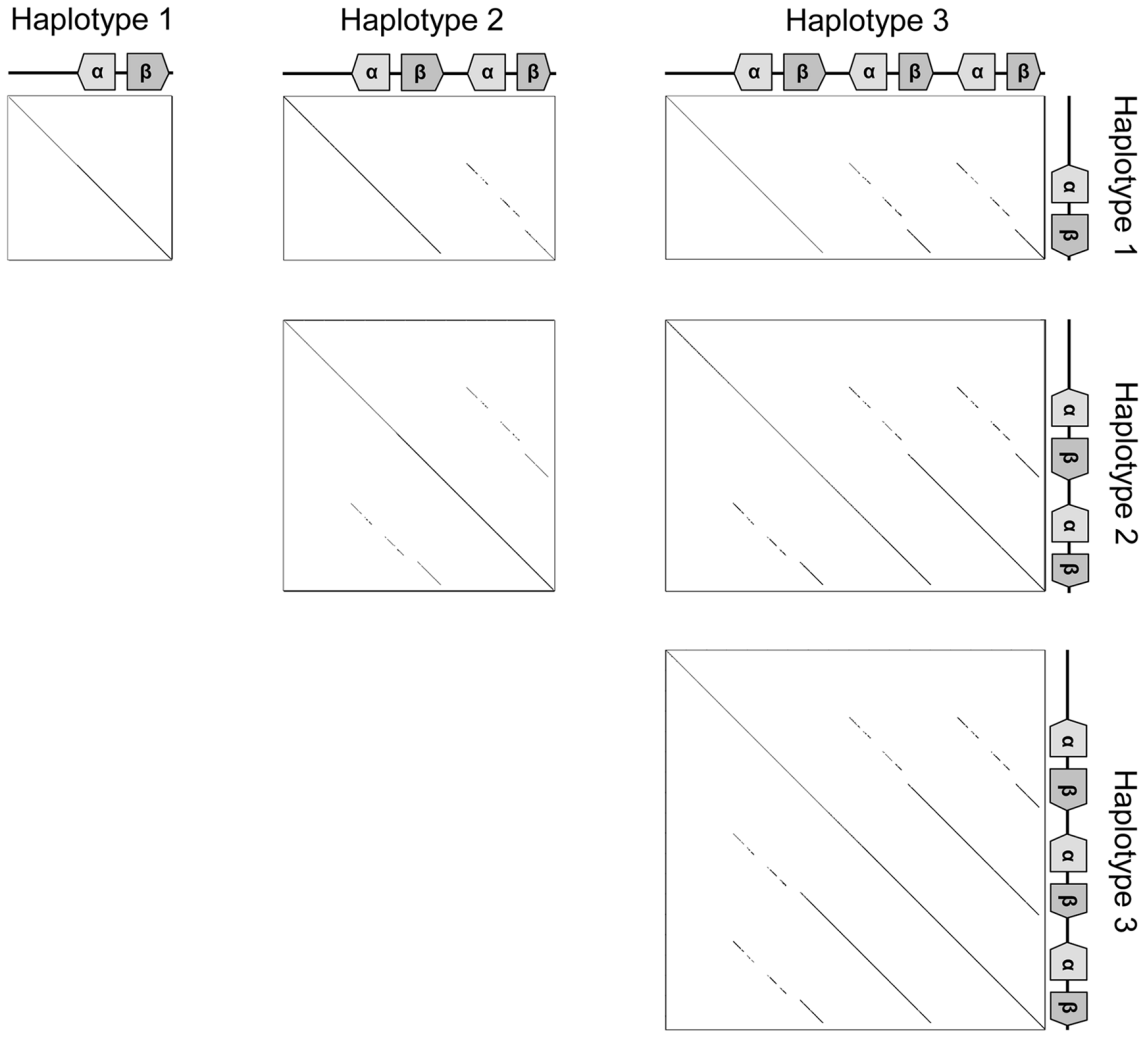
Dot-matrix analysis between three haplotypes of MHC class II region. Diagonal lines indicate regions where contiguous sequences align.

A dot-matrix analysis of exons and introns revealed that the *DAA2-DAB2* locus in HP3 was more similar to the *DAA3-DAB3* locus than the *DAA1-DAB1* locus (Figure 4). A remarkable feature was observed in the DAB1*01 allele in HP1: its 3′-terminal region was identical to that of the *DAB3* locus, whereas its 5′-terminal region was identical to that of the *DAB1* locus.

### Phylogenetic Analysis of the Japanese Crested Ibis MHC Class IIB Gene

**Figure 5:**
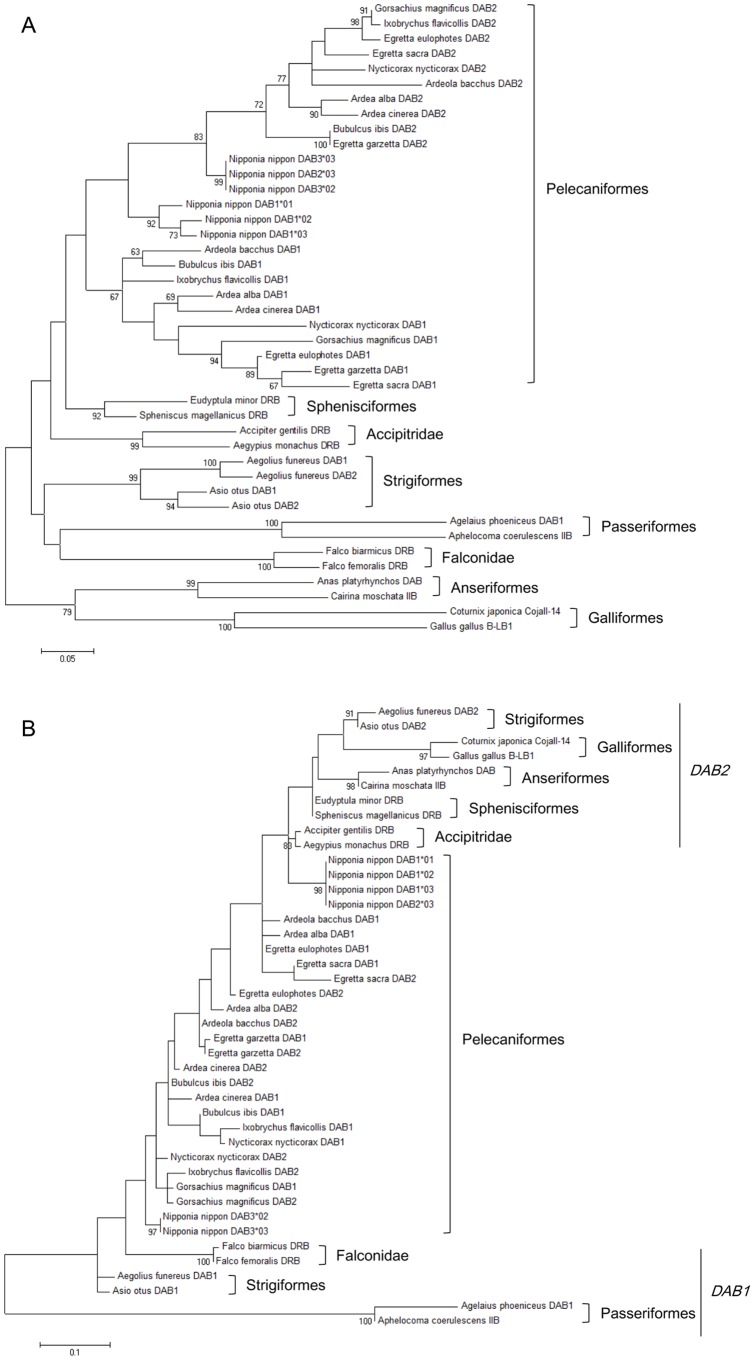
Maximum-likelihood tree with MHC-IIB exon 2 or partial exon 3 sequences from *Nipponia nippon* and other bird species. The best-fitting nucleotide substitution model for each codon position was evaluated using Find Best DNA/Protein Models (ML) in MEGA version 5.2 [28] according to the Akaike information criterion. (A) The tree of exon 2 was constructed by using a Kimura 2-parameter model with gamma distribution in MEGA. (B) The tree of partial exon 3 was constructed by using a Tamura 3-parameter model with gamma distribution in MEGA. Bird species used for the analyses were shown in Table S3. In both analyses, bootstrap values were evaluated with 1000 replications. Bootstrap values>60 are shown in this tree. Branch lengths represent the number of changes per site.

Phylogenetic relationships of MHC-IIB genes within 11 Pelecaniformes as well as among eight avian orders were analyzed using exon 2 or partial exon 3 sequences. The maximum-likelihood tree constructed using MHC-IIB exon 2 sequences showed that all Pelecaniformes formed one cluster separated from other avian orders (Figure 5a). The Japanese Crested Ibis belongs to family Threskiornithidae; the other 10 Pelecaniformes belong to family Ardeidae. In the Ardeidae, two MHC-IIB loci, *DAB1* and *DAB2*, were identified on the basis of differences in the length of intron 1 [27]. Within the Pelecaniformes cluster, three alleles (DAB3*02, DAB2*03, and DAB3*03) of the Japanese Crested Ibis were included in the *DAB2* subclade. Although the other three *DAB* alleles (DAB1*01–*03) were grouped into independent subclade, this branching was ambiguous because of a low bootstrap value (<60). The maximum-likelihood tree with partial exon 3 sequences was largely different from that with exon 2 sequences (Figure 5b). Among seven avian orders except for Pelecaniformes, two MHC-IIB lineages (*DAB1* and *DAB2*) were observed [6]. Within Pelecaniformes Ardeidae, partial exon 3 sequences tended to cluster together within species and did not show gene-specific cluster [27]. In contrast, four alleles (DAB1*01–*03 and DAB2*03) and two alleles (DAB3*02 and DAB3*03) of the Japanese Crested Ibis were grouped into two independent subclades separated from other Pelecaniformes. Phylogenetic relationships with MHC-IIB exon 2 and partial exon 3 sequences suggested that evolution of the MHC-IIB gene in *N. nippon* might be different from other Pelecaniformes.

### Genetic Diversity of MHC Class II Region among Five Founders

**Figure 6:**
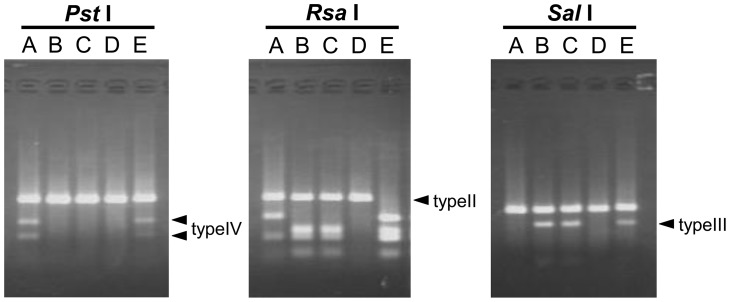
Genotyping of MHC class II gene regions by PCR-RFLP. A 279-bp fragment of MHC IIB exon 2 was amplified from five founder genomes (A–E). PCR products were digested with *Pst*I, *Rsa*I, or *Sal*I and digested fragments were analyzed by 3% agarose gel. Non-digested fragments with *Rsa*I, *Pst*I-digested fragments, and *Sal*I-digested fragments represented type II (DAB1*01 in HP1), type IV (DAB1*02 in HP2), and type III (DAB1*03 in HP3), respectively (Table 1 and Figure 1).

The current *N. nippon* population in Japan originates from only five founders. To investigate the genetic diversity of the MHC class II region among these founders, their MHC genotypes were examined by PCR-RFLP (Figure 6). Non-digested fragments with *Rsa*I, *Pst*I-digested fragments, and *Sal*I-digested fragments represented type II (DAB1*01 in HP1), type IV (DAB1*02 in HP2), and type III (DAB1*03 in HP3), respectively (Table 1 and Figure 1). The genotypes of founders A, B, C, D, and E were estimated to be *hp1/2*, *hp1/3*, *hp1/3*, *hp1/1,* and *hp2/3*, respectively.

**Figure 7:**
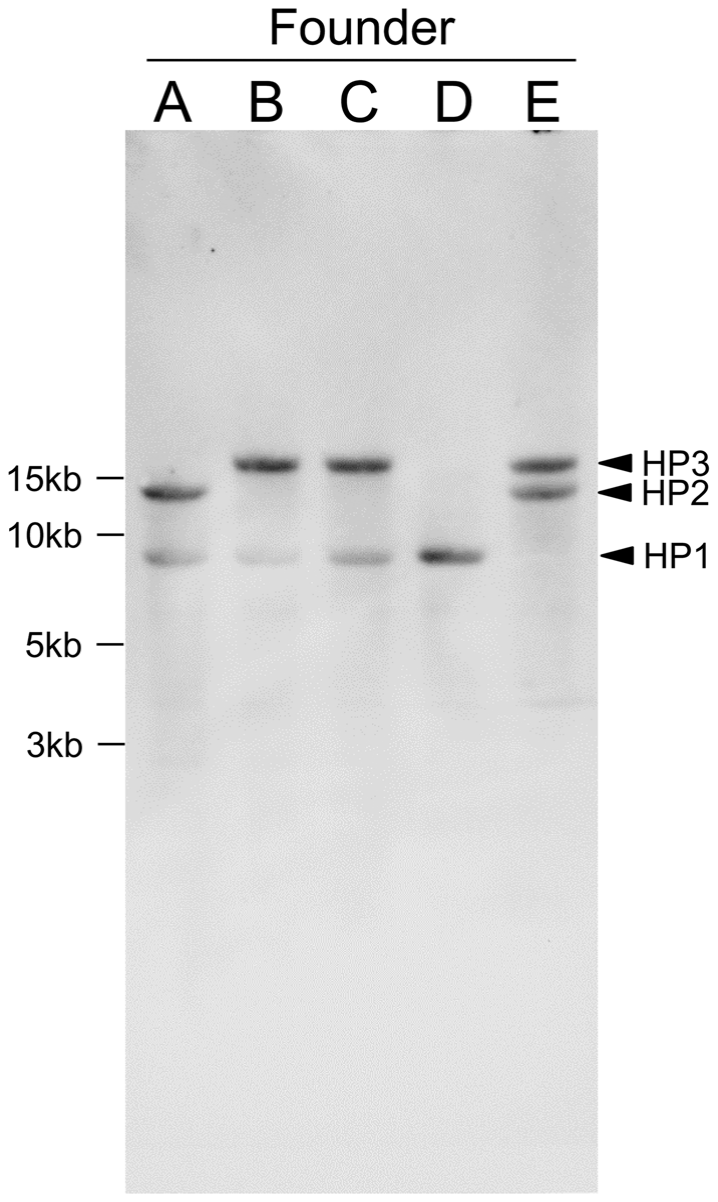
Detection of MHC class II gene regions by Southern blotting. Genomic DNA from five founders (A–E) was digested with *Bam*HI and *Eco*RI and hybridized with a mixture of three probes (MHC IIA exon 3, MHC IIB exon 2, and MHC IIB exon 3). Three bands of approximately 8, 13, and 18 kb represented HP1, HP2, and HP3, respectively.

The MHC genotypes of the founders were also analyzed by Southern blotting (Figure 7). Three bands of approximately 8, 13, and 18 kb were detected that corresponded to HP1, HP2, and HP3, respectively. The genotypes indicated by Southern blotting were the same as those determined by PCR-RFLP.

In four founder genomes (A–D), both of the alleles at the *DAB1* locus were individually amplified. Primers DAA-F1 and DAB*01-R1 were used for DAB*01, and primers DAA-F1 and DAB*02,03-R1 were used for DAB1*02 and DAB1*03. The DAB1*02 and DAB1*03 alleles in founder E were amplified from isolated lambda phage clones. The resulting PCR products were directly sequenced and sequences (approximately 1 kb) from exon 2 to exon 4 were compared. Four DAB1*01 sequences from founders A–D were completely identical, as were two DAB1*02 sequences from founders A and E and three DAB1*03 sequences from founders B, C, and E. These results strongly suggested that the founder population possessed only three MHC class II haplotypes. Moreover, no additional hybridizing bands besides the three haplotype bands were detected in the Southern blotting, suggesting that the MHC class II region was a single locus in the Japanese Crested Ibis genome.

## Discussion

The Japanese Crested Ibis is a critically threatened and internationally conserved species belonging to order Pelecaniformes, an avian lineage that is highly divergent from order Galliformes. We isolated genomic clones encompassing MHC-IIB genes from the *N. nippon* genomic libraries and constructed three contigs covering the MHC class II regions by genome walking (Figure 1). These contigs represented 3 different haplotypes of MHC class II regions. Our sequencing data revealed that the MHC class II genomic structure in *N. nippon* was largely different from that of chicken. The MHC-IIB gene was flanked by the MHC-IIA gene and the MHC IIA/IIB gene pair was located between *COL11A2* and *BRD2* in *N. nippon*, whereas the chicken MHC-B contained no MHC-IIA gene (*BLA*) and two MHC-IIB genes (*BLB1* and *BLB2*) located on both sides of the *Tapasin* gene [7]. We did not detect a *Tapasin*-like gene in cloned fragments. The gene order *COL11A2*-MHC-IIA, MHC-IIB-*BRD2* in *N. nippon* was more similar to that in humans than to that in chicken [7], [8]. In the duck MHC, a single MHC-IIA gene was located next to five MHC-IIB genes, whereas the genes on both sides of the MHC IIA/IIB cluster are unknown [16]. In contrast with the duck MHC, in which only the MHC-IIB gene was duplicated, gene duplications occurred as a unit with MHC-IIA/IIB gene pairs in *N. nippon*.

Our results for polymorphism of MHC-IIB exon 2 (Table 1), cloning (Figure 1), and Southern blotting (Figure 7) suggested that the MHC class II region was a single locus in the Japanese Crested Ibis genome, although we cannot exclude the possible existence of other MHC class II loci with low sequence homology to those presented here. These results are similar to findings in duck [16] but different from those in chicken, in which additional MHC class II genes were found in the MHC-Y region [9], [10]. These combined results suggest that there is large variability in MHC genomic organization among avian species at the order level.

The three haplotypes of MHC class II regions in *N. nippon* had different copy numbers of MHC IIA/IIB gene pairs (Figure 1). In MHC-IIA genes, three alleles (DAA1*01–*03) at the *DAA1* locus encoded identical proteins, as did another three alleles (DAA3*02, DAA2*03, and DAA3*03) (Figure 2). However, three MHC-IIB alleles (DAB1*01–03) contained 12 polymorphic amino acid residues within exon 2 (Figure 3). MHC-IIB genes were apparently more polymorphic than MHC-IIA genes. In contrast with the *DAB1* locus, the alleles DAB3*02, DAB2*03, and DAB3*03 had an identical exon 2. Haplotypes 2 and 3 might have been produced by duplication and/or gene conversion during a relatively recent period in evolutionary time. Moreover, the characteristic of DAB1*01, in which its 5′- and 3′-terminal regions were identical to those of *DAB1* and *DAB3* loci, respectively (Figure 4), might indicate that DAB1*01 was produced by recombination between the *DAB1* and *DAB3* loci. Copy number variations of MHC-IIB gene has been reported in certain bird species [11], [30]–[32]. These results might indicate that gene duplication, gene loss and/or gene conversion are operating at relatively high rate within a single bird species

Our phylogenetic analyses showed that Pelecaniformes formed one cluster separated from other seven avian orders (Figure 5a), suggesting that MHC-IIB genes of *N. nippon* were closely related to those of the other Pelecaniformes from family Ardeidae that were examined in this study. Two MHC-IIB loci, *DAB1* and *DAB2*, with longer and shorter intron 1 lengths, respectively, were detected in Ardeidae [27]. The three *DAB1* alleles in *N. nippon* possessed a longer intron 1 (661 bp) than did DAB3*02, DAB2*03, and DAB3*03 (285 bp). The latter three alleles were grouped into the *DAB2* subclade, but DAB1*01–*03 were not in the *DAB1* subclade in the maximum-likelihood tree with exon 2 sequences, suggesting that evolution of the MHC-IIB gene might differ among families within the same order. Moreover, the relationship between two MHC-IIB loci (*DAB1* and *DAB2*) in Pelecaniformes Ardeidae was obviously different from that in Strigiformes (Figure 5). Although the maximum-likelihood tree with partial exon 3 sequences suggested that the *DAB3* and *DAB1* loci in *N. nippon* might represent two MHC-IIB lineages (*DAB1* and *DAB2*) respectively (Figure 5b), the genome structures emerged that three *DAB* loci might be a cluster in the same lineage (Figure 1). To elucidate the long-term evolutionary history of the avian MHC, more data on genome structure of MHC from a wide diversity of bird species are apparently needed.

As the current Japanese population of *N. nippon* originates from a small number of founders, polymorphism among these founders should largely limit the genetic diversity of progeny. We identified only three kinds of MHC class II haplotypes among the five founders. In the Chinese population of *N. nippon*, five alleles of MHC-IIB exon 2 were detected [33]. Three out of five alleles were not observed among the founder birds in Japan. Microsatellite markers in the Chinese population contained two to five alleles, and only two haplotypes were detected in mitochondrial DNA control regions [34]–[36]. These findings suggested that the Chinese population experienced a severe evolutionary bottleneck. There were two to three alleles of microsatellite markers and haplotype numbers of 202-bp sequences containing multiple SNP sites in the five founders of the Japanese population [37], [38]. These results strongly suggested that the number of alleles/haplotypes in the current Japanese population are three or fewer in most genomic loci and that genetic diversity is extremely low. The finding of lower genetic diversity in the Japanese population compared with the Chinese population is reasonable because the founders of the Japanese population originated from China.

To our knowledge, this is the first report of MHC class II genomic organization in Pelecaniformes. Our results revealed that the MHC class II genomic structure of the Japanese Crested Ibis was largely different from that of Galliformes or Anseriformes. Moreover, the fact that five founders possessed only three kinds of MHC class II haplotypes strongly suggested that the genetic diversity of the MHC region in the Japanese population is extremely low. The recovery of a large population from a small number of founders with low genetic diversity will be a significant challenge. If successful, the process might provide a good model for investigating the expansion of genetic diversity in a closed population through mutations and recombination events. The structure of the MHC class II region presented here will provide valuable insights for future studies on the evolution of the avian MHC and for conservation of the Japanese Crested Ibis.

## Supporting Information

Figure S1 **Schematic of polymerase chain reaction (PCR) screening for the lambda phage library.** In the first screening, lambda phages from the primary library were plated on 90-mm plates at 20,000 pfu/plate. Twenty-five plates were used for the screening of 500,000 independent clones. After clear plaques appeared, the plates were overlaid with 3 mL of SM buffer and stored at 4°C overnight. Next, phage solutions were individually collected into 25 tubes (first phage pool containing 20,000 clones). Phage solutions were directly used as template DNA for PCR. The positive first phage pool was selected by PCR using KOD-FX Neo DNA polymerase (Toyobo). In the second screening, phages from the positive pool were plated on four plates at 80,000 pfu/plate. After clear plaques appeared, NZY agarose gel with plaques was cut into 1- cm^2^ blocks (containing approximately 1600 clones/block). Each block was transferred into a 24-well plate with 400 µL of SM buffer. The second screening used 96 blocks. Positive blocks were selected by PCR, further divided into 16 sections (0.0625 cm^2^ containing approximately 100 clones each), and transferred into 1.5-mL tubes with 100 µL of SM buffer. Positive 0.0625 cm^2^ blocks were selected by PCR. In the third screening, phages were plated at 1,250 pfu/plate (n = 2 plates). Positive 0.0625 cm^2^ blocks (containing several clones) were selected in the same manner as for the second screening. In the fourth screening, phages from the positive pool were plated 150 pfu/plate (n = 1 plate). After clear plaques appeared, 20 single plaques were transferred into 1.5-mL tubes with 100 µL of SM buffer. A single positive plaque was selected by PCR.
(PDF)

Figure S2 **Partial sequence of** ***collagen-type XI α-2-like*** **gene in the** ***Nipponia nippon*** **MHC class II region.** B, P, Sc, and X represent restriction sites used for subcloning of *Bam*HI, *Pst*I, *Sac*I, and *Xho*I, respectively. Solid bars and arrows below the map indicate the location of isolated lambda phage clone and sequenced regions, respectively. Gap regions are shown as “N.” A BLAST search against the human genome + transcripts database revealed that this partial sequence was homologous to *collagen-type XI α-2 transcript*.
(PDF)

Table S1 **List of primers used for polymerase chain reaction analysis.**
(XLS)

Table S2 **Polymorphism of MHC-IIB exon 2 in two founders and 20 progeny of Japanese Crested Ibis.**
(XLS)

Table S3 **Bird species used in phylogenetic analysis.**
(XLSX)
