# Supplementary figures and images for: Structure and Polymorphism of the Major Histocompatibility Complex Class II Region in the Japanese Crested Ibis, Nipponia nippon
Source: PLoS One. 2014 Sep 23;9(9):e108506. doi: 10.1371/journal.pone.0108506 (PMC4172706; doi:10.1371/journal.pone.0108506)

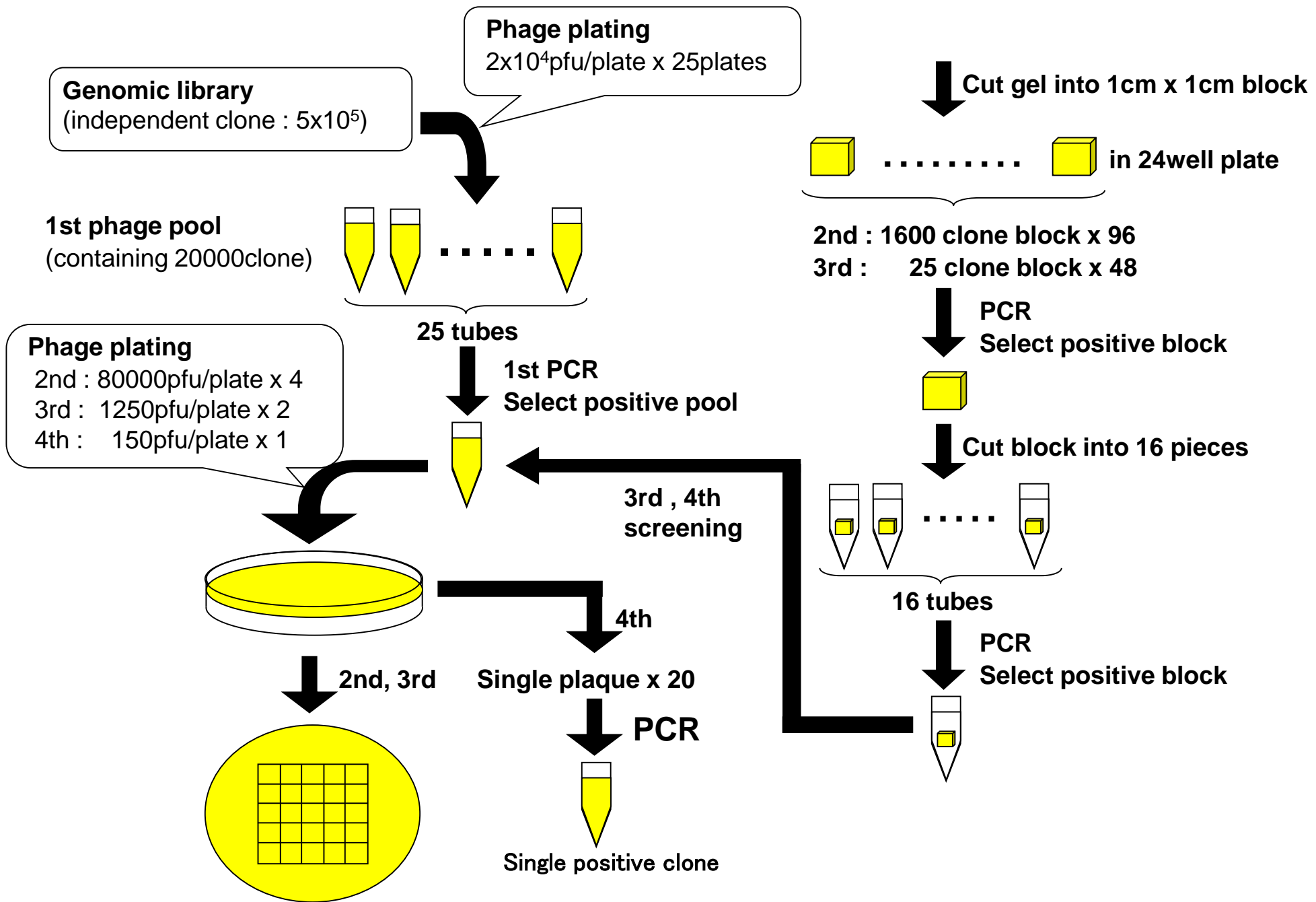

Supplement: Figure S1 — Schematic of polymerase chain reaction (PCR) screening for the lambda phage library. In the first screening, lambda phages from the primary library were plated on 90-mm plates at 20,000 pfu/plate. Twenty-five plates were used for the screening of 500,000 independent clones. After clear plaques appeared, the plates were overlaid with 3 mL of SM buffer and stored at 4°C overnight. Next, phage solutions were individually collected into 25 tubes (first phage pool containing 20,000 clones). Phage solutions were directly used as template DNA for PCR. The positive first phage pool was selected by PCR using KOD-FX Neo DNA polymerase (Toyobo). In the second screening, phages from the positive pool were plated on four plates at 80,000 pfu/plate. After clear plaques appeared, NZY agarose gel with plaques was cut into 1- cm2 blocks (containing approximately 1600 clones/block). Each block was transferred into a 24-well plate with 400 µL of SM buffer. The second screening used 96 blocks. Positive blocks were selected by PCR, further divided into 16 sections (0.0625 cm2 containing approximately 100 clones each), and transferred into 1.5-mL tubes with 100 µL of SM buffer. Positive 0.0625 cm2 blocks were selected by PCR. In the third screening, phages were plated at 1,250 pfu/plate (n = 2 plates). Positive 0.0625 cm2 blocks (containing several clones) were selected in the same manner as for the second screening. In the fourth screening, phages from the positive pool were plated 150 pfu/plate (n = 1 plate). After clear plaques appeared, 20 single plaques were transferred into 1.5-mL tubes with 100 µL of SM buffer. A single positive plaque was selected by PCR. (PDF) [file pone.0108506.s001.pdf]
