# Supplementary material for: Structure and Polymorphism of the Major Histocompatibility Complex Class II Region in the Japanese Crested Ibis, Nipponia nippon
Source: PLoS One. 2014 Sep 23;9(9):e108506. doi: 10.1371/journal.pone.0108506 (PMC4172706; doi:10.1371/journal.pone.0108506)
Supplement: Figure S2 — Partial sequence of collagen-type XI α-2-like gene in the Nipponia nippon MHC class II region. B, P, Sc, and X represent restriction sites used for subcloning of BamHI, PstI, SacI, and XhoI, respectively. Solid bars and arrows below the map indicate the location of isolated lambda phage clone and sequenced regions, respectively. Gap regions are shown as “N.” A BLAST search against the human genome + transcripts database revealed that this partial sequence was homologous to collagen-type XI α-2 transcript. (PDF) [file pone.0108506.s002.pdf]

Haplotype 1

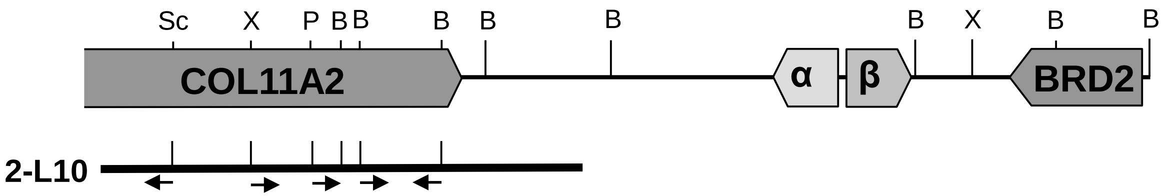

GCGAAACAAACCCCTCTCCTAGTTATCATCCATTCTAATTGTCAATTGGTGTGTGAGTGGTTCATCTGGTATTGAAAGCATACTTAGGGAGGTTCAACCTCTGCCCACGTTTCATTAAATAAAAT TGCTCCCTCTAGAAAAA
GAATAACCGTTGGATTTGCAGGCCCGGGCGTGAAGAGCCCCAAGGTAAGTTTTGAGCAGCGTTAAAGGGGGGACTGTGATCTCCCCCCTCCCCACACCCCTCTCCTGGGGGGTGGGGGGGCTCCC TGGGGGGGTGGGGGGG
CCGATGGACCATCCATCACCCCCCCTCCACCTCCTCCCGCAGGGTCCCAGGGGTCAGCGGGGACCACGAGGAGCCACCGGCAAGTCAGGGGCAAAGGTCAGTCTTTGGGGGGGGGGGGGGCGGGC TCACCCTGGCCCTTGTG
CGTGTACAATCGCACACGCGCGTGACAGCTCTGGCACCGACGTGCGCCGGAGCTGGGCACCCGCTGGGCACCCGCAAACCTTCCTCGCTCCCTCAGGGCACGTCTGGCAGCGATGGCCCCCACGG CCCCCCAGGTGAACGGG
TAAGTGGGCGTCCCAGCCTGGATTTAGAGGGGGGGGTGGGGGTCCCAGCTTGCACGAGCACCCTGGTGCCATCCCCACCAGCCACCCACCGCACTGTGCCAAGCTCTGGGACCCCCCCCCCAGTGC CCGCCATCACCCAGACC
CCCAGCTCCCTCCCGCCCGGCGCACGCAGCTCCGTCTCCCCTCGCGCCGGGGTGGGGGGGGGGTTGGTGATGCTGTGCTAACGAGCTCNNNNNNNNNNNNNNNNNNNNNNNNNNNNNNNNNNNNNNN NNNNNNNNNNNNNNNNN
NNNNNNNNNNNNNNNNNNNNNNNNNNNNNNNNNNNNNNNNNNNNNNNNNNCTCGAGCTGACGCCCTCCTCCCTCCATCTTCCCCCAGGGCTCCCCTGGGGAGAGAGGTGGCCCGGGGCAAGGTGGTC CCGTTGGCCCCCCAGGA
CGACCAGGACCACAGGGACCACCGGGCCCTGCCGGAGAGAAAGGAGTTCCCGTGAGTGCTCAGGGACATCCCCCTCGCATCCCGCAGGGAAAGGCGGCGGGTCCAGGAGGGGTGCAAGGGGGTGG GTGCTTGCACCCCTCAC
CCCCCCCCTTTTCACATCCCTCTAGGGTGAGAAGGGCCCCATGGGTCCCGCTGGCCGGGATGGCGTTCAGGGTCCTGTGGGTTTACCTGGCCCAGCCGGCCCCCCCGGCGGCCCCGGGGAGGACG GTGACAAGGTGAGGTCC
TACCCTGCCCCGGTGTCACCGTGCGCCCCGATGCACTGGGGCTGAACACCCTTCTTCTCCCCTCCAGGGTGAGGTGGGCGAGCCGGGACAGAAGGGCAGCAAAGGCAACAAGGGCGAGCACGTGA GTGTGATGGGTGTCCCC
CCCTCCCCATACCTTGTCCCTGCTGTCCCCAGGGGCTGTCCCCAGCCTCACTGACCCCTTCCCTCCCTCACAGGGTCCTCCTGGTCCCCCTGGGCCCATCGGGTCAGTCGGGCAGCCCGGAGCCG CTGTGAGTATCCCAGAG
GCAGGGCAACGTCACGGTCTGTGGGCGCGATGTGGGACGGAAGGCTCCTGTGCCACACCTGCGGCCACACGCAGTAGGGGACGGTCTCAGCTAGGAGACGTCCCTGTCCTCNNNNNNNNNNNNNN NNNNNNNNNNNNNNNNN
NNNNNNNNNNNNNNNNNNNNNNNNNNNNNNNNNNNNNNNNNNNNNNNNNNNNNNNNNNNNNNNNNNNNNNNNNNNNNNNNNNNNNNNNNNNNCTGCAGGGACCCCAGGGACCCCCTGGAGG TGTTGGGAACCTGGGAC
CTCCTGGTGAGAAGGTACGTCCCCGGACATCGGGGTTCGCTCCCCGGGAGCCGCCCTAGCGGGGTGCAGAGGTGGGGTGAGGCCGTGGGGCCGTAGTGGGTCTATGGGACAAGGAACCAATCCGG AGGGCTTGGTCCCAGAG
CGGGGACAAGGTGGCACGGTGCGGGGAGGGTTTGACACCTCTCTGTTCCCGTTTCAGGGGGACCCCGGTGAATCGGGAGCCCCGGGAATCCAAGGGGAGCCAGGAGTCAAGGTGAGTGTCAGCAG GGACACCCCGCTTTCTG
TCCCCGTCCCCTGGCTGGCCCTGCCGGCTGCTGACCCCATCTTGCACCGCAGGGCCCACGAGGAGAGCGAGGGGAGAAGGGAGAAGCCGGAGCTGCCGGTGCTGCTGGGCCACCTGGAGGGAAAG GGCCCCCCGGCGACGAC
GGGCCCAAGGGCAACCCCGTGAGTGCCGTGTCCCCACCCCAGGATGCAGGGGACACGGGGAGGGAGCAGGAGGGGTTCGCTCCGGGAAGGCGTTGGGACCCCTTGGAGGGGAGGTCCAGGGTCCC TCAGGCTAGCAGCTCGC
TCTGTTTCAGGGTCCCGTTGGCTTCCCAGGCGATCCCGGACCCCCAGGTGAAATGGGTCCCAGGGTGAGTGTCGCATCCCACCCCCTCTGGGCTTCTACTGGAGGGGGCTTCCCTGGGAATGGCG TTGGGATTTGGGGGTGT
GTGCGGGACCTTGTGCTGACTCCAGGTTTTCCCTTCCCGACAGGGCCAGGATGGCGCCAAGGGCGAGCGTGGGGAGGACGGCGAGCCCGGGGAGCCGGTGAGTGCCCCGTTCCGGCACGTCCCCC CCTCACCATCGCTCTGG
GCAGGGGTGTCTCGACCAGATCCCCCTGCGAGGTTTGGGACTGGATTAACTCCACTGCTTCAGGAATGTCAGGGAGTGGGGAGCGCAGCCTGTCCCCCCTTCCGCGGCCATTGGATCTGGTCCCC CTGGTCCTGGGATCCNN
NNNNNNNNNNNNNNNNNNNNNNNNNNNNNNNNNNNNNNNNNNNNNNNNNNNNNNNNNNNNNNNNNNNNNNNNNNNNNNNNNNNNNNNNNNNNNNNNNNNNNNNNNNNNNNNNNNNNNNNN NNNNNGGATCCCTGGTG
CTACTGGACCCATCGGCCCCGCCGGCCCCCCCGGACTTCCCGTGAGTGCTCCCCGTGTGCTATCGGGTCCCTCCCTCTCCTCGCCCCGCTGCTCGCTCCCCCTCTCTCCTCCTTGTGCCTGGGCT CCCAGTTTCCCTCCCTG
CGTTTGCGGGGGCGGGGGGGCTGCTGTCTCCCCACCCCACTTTGCCAGCCCCAGTCGCTGACAGACCTGGTTTTGCTTTGCAGGGCCCAGCTGGTCCCAAAGGTGCCAAGGGAGCCATGGTAAGC GCCGAGGCGGTGCCCCC
ACCTTGCCACCACCTTCAGTCCTGCCCAAGGCTGACCTCTGTGAGTGTCTCTTTCCTCTAGGGTCAAGCAGGACCCAAAGGTGAACGTGGACCCCCAGGACCCCCTGGACACCCGGTGAGTGGCT CAAGCGTTGGCACCAGG
GAAGGAATGACACCGGGGTGGGGGGATGTAGGAACAGCTCCTCGGTAGGTAAAATGACACCCAGGAATGGACGTGGAGGGATGTCTTCTATCTCAAAGCCTGAGCACGGGGTGGTTTGGGGCCCT GGGGAGTGGGGGCTTCG
CAGCCCCCCATGCTCGGGTGCTGACAGTGGTGTGCCTGCCACAGGGTCCCCCTGGGGAGGTGATCCAGCCCCTGCCAATCCAGCTGCCCAAGAAGAGCAAGCGCTCCATTGACGCCAGCANNNNN NNNNNNNNNNNNNNNNN
NNNNNNNNNNNNNNNNNNNNNNNNNNNNNNNNNNNNNNNNNNNNNNNNNNNNNNNNNNNNNNNNNNNNNNNNNNNNNNNNNNNNNNNNNNTAGAGGTGAGCACGCCCCACCTGGAGCAGCTCCCGCT GCTGGACGTGCGCGTCA
TGGACTTCGGCGAGCCCGGCCAGAGGTTTGGGTTCGAGGTGGGCCCAGTCTGTTTCCTCGGCTAGGACCAAACCGTCACCCTCCTTGCCACACCCTGGCCTCCAGTGGGGCAAACCTTGCCTCCT CGCATCCCCCCACTGCC
TCCGAGCGGCGAAGCAGCAAGACCCGCCTCCCCCTCGCATCACGTCACCCACCCGCTGGTGCGAGGAGCTGCGGTGCCAGCCCTGCCCACACGCACCGAGACCGACGCGCCGCCGCTGCCGCCCC CACGGGGGCCAGGGTCT
GGAGAGATGCCAAAGAGACTTTAACGCTTAGAATAAATCTATATTTTAATATGTAGAGAGGCAGAGAGGAAAATTTTTAACGGATCCCCCCCTCGGGGAGCTATGGCTGGGGAGAGGGGGCCACA CTCACCACCGGTCCTGG
TGGCCCAGCCTGGCCTGTGGTACCCGGTTTGCCTTGTTCTCCCTGCACAGGCGAGAGGACAAGGTGAGTCCCACCGGTCTGGAGTCGGTGCCGCCGGTGGCAGCGCACACCCCGGGCAGCTCCCC TTGAACCATGGGCACAG
TGACATGTCCCAGTCCCCTCCCGGTGTGACAGTCAGACCCTCACCCCTCCTCTGCGCTTTGACCCCGGGCATGGGATCC
